# PHYSICAL AND MENTAL HEALTH OF GRANDPARENTS CARING FOR GRANDCHILDREN IN CHINA: A SYSTEMATIC REVIEW

**DOI:** 10.1093/geroni/igae098.2748

**Published:** 2024-12-31

**Authors:** Shuangshuang Wang

**Affiliations:** Southwest Jiaotong University, Chengdu, Sichuan, China (People’s Republic)

## Abstract

Guided by role strain and role enhancement perspectives, this study systematically reviewed the effects of providing grandchild care on the physical and mental health of Chinese grandparents, aiming to identify research gaps and offer insights for future studies and the enhancement of Chinese grandparents’ well-being. Utilizing a thematic search method, we conducted searches in PubMed, Web of Science, CNKI, Wan Fang Data, and CQVIP databases for relevant literature spanning from January 1st, 2012, to March 1st, 2024. Ninety-one articles were ultimately included, and their content was analyzed to systematically review the impacts on physical and mental health. Factors such as grandparents’ gender, age, and residential areas affected their participation in intergenerational care. Mental health outcomes primarily focused on depressive symptoms, cognition, loneliness, and life satisfaction, while physical health was assessed through self-assessed health, daily-life abilities, and instrumental self-care abilities. However, the literature lacked consensus on the effects of providing grandchild care on Chinese grandparents’ physical and mental health. Negative outcomes mainly due to role conflicts, high care demands, feelings of isolation, while positive outcomes were achieved through social more social activities, companionship, and family network support. In conclusion, Chinese grandparents’ well-being appears to be influenced by a multitude of factors, with current exploration in its nascent stages. Opportunities for improvement lie in data collection methods, research design, and areas of investigation. Further research is warranted to better understand the complexities of this relationship and to develop interventions aimed at promoting the health and well-being of Chinese grandparents.

